# Supplementary material for: Method development and characterisation of the low-molecular-weight peptidome of human wound fluids
Source: eLife. 2021 Jul 6;10:e66876. doi: 10.7554/eLife.66876 (PMC8260221; doi:10.7554/eLife.66876)
Supplement: Supplementary file 1. [file elife-66876-supp1.docx]

Supplementary File 1 Summary of mass spectrometry results for the three types of samples

|  | **Samples** |  | | **Peptides** | |  | | **PSM** | |  | | **Proteins** | |  | | **MeOx** | |  | | **NQ** | |  | | **Data depiction** | |  |
| --- | --- | --- | --- | --- | --- | --- | --- | --- | --- | --- | --- | --- | --- | --- | --- | --- | --- | --- | --- | --- | --- | --- | --- | --- | --- | --- |
| **Citrate plasma** | 3 |  | | 770 | |  | | 2827 | |  | | 65 | |  | | 126  (4.5%) | |  | | 87  (3.1%) | |  | | F3  SD1 | |  |
| **Acute wound fluids** | 5 |  | | 7809 | |  | | 47228 | |  | | 373 | |  | | 10243  (21.7%) | |  | | 3009  (6.4%) | |  | | F1-4  SF2, SD2 | |  |
| **Dressing extracts** | 6 |  | | 10789 | |  | | 52091 | |  | | 418 | |  | | 5059  (9.7%) | |  | | 1774  (3.4%) | |  | | F5-8, F5FS1  SF3, SD3 | |  |
|  |  | |  | |  | |  | |  | |  | |  | |  | |  | |  | |  | |  | | **Proteins** | |

Total numbers of unique samples, identified unique peptides, peptide spectrum matches (PSM), and unique proteins. Furthermore, total numbers and percentages of peptides with post translational modifications (MeOx: methionine oxidation; NQ: deamidation) are indicated. Finally, it is indicated in which figures (F), figure supplements (FS), supplementary files (SF), and supplementary datasets (SD) the results of the sample analyses are shown.
