# Supplementary material for: Method development and characterisation of the low-molecular-weight peptidome of human wound fluids
Source: eLife. 2021 Jul 6;10:e66876. doi: 10.7554/eLife.66876 (PMC8260221; doi:10.7554/eLife.66876)
Supplement: Supplementary file 2. [file elife-66876-supp2.docx]

Supplementary File 2 Identified peptides and their average length from five acute wound fluids

|  |  | **WF1** |  | **WF2** |  | **WF3** |  | **WF4** |  | **WF5** |
| --- | --- | --- | --- | --- | --- | --- | --- | --- | --- | --- |
| **Number of peptides** |  | 3876 |  | 4271 |  | 2932 |  | 2649 |  | 3435 |
| **Number of proteins** |  | 188 |  | 222 |  | 150 |  | 143 |  | 196 |
| **Average length (Da)** |  | 1414.70 |  | 1367.43 |  | 1463.87 |  | 1516.11 |  | 1373.36 |
| **Average number of AA** |  | 12.69 |  | 12.28 |  | 13.26 |  | 13.68 |  | 12.28 |

Total numbers of identified unique peptides, proteins, and the average length and number of amino acids (AA) detected in five acute wound fluids. Results are combined data of 4 injections per sample.
