# Supplementary material for: Method development and characterisation of the low-molecular-weight peptidome of human wound fluids
Source: eLife. 2021 Jul 6;10:e66876. doi: 10.7554/eLife.66876 (PMC8260221; doi:10.7554/eLife.66876)
Supplement: Supplementary file 3. [file elife-66876-supp3.docx]

Supplementary File 3 Identified peptides and their average length from six dressing extracts

|  |  | **Low inflammation** | | | | |  | **High inflammation** | | | | |
| --- | --- | --- | --- | --- | --- | --- | --- | --- | --- | --- | --- | --- |
|  |  | **1** |  | **2** |  | **3** |  | **4** |  | **5** |  | **6** |
| **Number of peptides** |  | 3146 |  | 4863 |  | 4277 |  | 5103 |  | 1412 |  | 4929 |
| **Number of proteins** |  | 179 |  | 228 |  | 200 |  | 170 |  | 88 |  | 175 |
| **Average length (Da)** |  | 1548.67 |  | 1591.19 |  | 1546.77 |  | 1589.84 |  | 1351.38 |  | 1668.49 |
| **Average number of AA** |  | 14.33 |  | 14.52 |  | 14.13 |  | 14.44 |  | 12.59 |  | 15.29 |

Total numbers of identified unique peptides, proteins, and the average length and number of amino acids (AA) detected in six dressing extracts.
